# Measurement of anti-DFS70 antibodies in patients with ANA-associated autoimmune rheumatic diseases suspicion is cost-effective

**DOI:** 10.1007/s13317-016-0082-1

**Published:** 2016-07-29

**Authors:** Simón Gundín, Juan Irure-Ventura, Esther Asensio, David Ramos, Michael Mahler, Victor Martínez-Taboada, Marcos López-Hoyos

**Affiliations:** 1 Hospital Universitario Marqués de Valdecilla-IDIVAL, Santander, Spain; 2 Inova Diagnostics, San Diego, CA USA

**Keywords:** Antinuclear antibodies, DFS-70, Indirect immunofluorescence, Cost-effectiveness, ANA-associated autoimmune rheumatic diseases

## Abstract

The presence of antinuclear antibodies (ANA) is associated with a wide range of ANA-associated autoimmune rheumatic diseases (AARD). The most commonly method used for the detection of ANA is indirect immunofluorescence (IIF) on HEp-2 cells. This method is very sensitive but unspecific. As a consequence, ANA testing on HEp-2 substrates outside a proper clinical specialist framework may lead to inappropriate referrals to tertiary care specialists and, worst case inappropriate and potentially toxic therapy for the patient. Among ANA, isolated anti-DFS70 antibodies represent a potentially important biomarker that can be clinically used to discriminate AARD from non-AARD patients in ANA IIF positive individuals. Therefore, their presence may avoid unnecessary follow-up testing and referrals. In our study, we investigated if the implementation of a new ANA workup algorithm allowing for the identification of anti-DFS70 antibodies is cost-effective through the reduction of both unnecessary follow-up testing and outpatient clinic visits generated by the clinical suspicion of a potential AARD. None of the 181 patients included with a positive monospecific anti-DFS70 antibody result developed SARD during the follow-up period of 10 years. The reduction in number of tests after ANA and anti-DFS70 positive results was significant for anti-ENA (230 vs. 114 tests; *p* < 0.001) and anti-dsDNA antibodies (448 vs. 114 tests; *p* < 0.001). In addition, the outpatient clinic visits decreased by 70 % (*p* < 0.001). In total, the adoption of the new algorithm including anti-DFS70 antibody testing resulted in a cost saving of 60869.53 € for this pilot study. In conclusion, the use of anti-DFS70 antibodies was clearly cost-efficient in our setting.

## Introduction

The presence of antinuclear antibodies (ANA) directed against intracellular antigens is associated with a wide range of disorders, including ANA-associated autoimmune rheumatic diseases (AARD). The most commonly used method for ANA detection in daily routine is the indirect immunofluorescence test (IIF) on HEp-2 cells. Since 1958, when this test was first described, it has revolutionized the diagnosis of AARD, especially that of systemic lupus erythematosus (SLE) and systemic sclerosis (SSc). The value of this test has been reinforced by the American College of Rheumatology and their recent task force recommendations, indicating that the IIF ANA method on HEp-2 cells should remain the screening test of choice [1]. However, one of the disadvantages of this test is its low specificity for AARD [2, 3], which is a major drawback when used in a low disease prevalence population. Up to 20 % of serum samples from healthy individuals (HI) have been reported to have a positive ANA test, the majority of them due to the presence of anti-DFS70 antibodies [4].

A landmark study on the clinical utility of the dense fine speckled pattern and anti-DFS70 antibodies showed that the DFS IIF pattern was found in 33.1 % of ANA-positive HI compared to 0.0 % of ANA-positive AARD patients (*p* < 0.0001), which significantly affects the diagnostic power and efficiency of the IIF assay. Regarding accurate pattern recognition, it is important to point out that the dense fine speckled pattern is not exclusive to the presence of anti-DFS70 antibodies, and appropriate interpretation and reporting of results are important because they could influence the referral of patients with a positive ANA, resulting in unnecessary tertiary care consultation. The discrimination between DFS and the so-called ‘quasi-homogeneous pattern’ might be a particularly challenging task for routine diagnostic laboratories [2], and inaccurate interpretation may have significant consequences.

Anti-DFS70-positive patients classified as non-AARD at the time of ANA testing will probably remain as such according to the studies that showed anti-DFS antibodies were more prevalent in HI than in patients with AARD and that anti-DFS-positive individuals did not develop AARD after clinical follow-up of 4 years [3]. Based on these observations, it has been suggested that the presence of isolated anti-DFS70 antibodies could be used as a biomarker to exclude the diagnosis of AARD, such as SLE [3–5].

It is quite obvious that ANA testing on HEp-2 cells outside a proper clinical specialist framework may yield a sizable portion of ANA-positive individuals without consistent evidence of AARD, purportedly leading to inappropriate referrals to tertiary care specialists, as well as anxiety in patients and physicians alike [3] and, perhaps, inappropriate and potentially toxic therapy [6].

Taking all this into account, and considering advances in autoimmunity research and the availability of new autoantibody assays, it is important to develop and implement novel test algorithms for ANA testing to support the diagnosis of AARD [7, 8].

Anti-DFS70 antibodies are directed against a co-activator of nuclear transcription, also known as p75, encoded by the *PSIP1* gene [9]. However, the primary target auto-antigen was previously identified as the lens epithelium-derived growth factor (LEDGF) [10]. The short name, DFS70, according to the IIF pattern (dense fine speckled) and the apparent molecular weight in immunoblot assays (70 kDa) is often used to refer to this antigen.

Anti-dense fine speckled 70 (anti-DFS70) antibodies were initially identified as an ANA IIF pattern from a patient with interstitial cystitis [11]; however, their presence is associated with various other conditions. The highest prevalence of these antibodies has been reported in patients with Vogt–Harada syndrome (66.7 %) [12], atopic dermatitis (AD, 30 %) [13, 14], followed by HI (10 %) [4, 9]. Their presence is associated with various chronic inflammatory disorders, cancer. Several studies showed that anti-DFS70 antibodies are common among ANA-positive individuals with no evidence of AARD.

To conclude, it is accepted that the presence of isolated anti-DFS70 antibodies could be taken as strong evidence against a diagnosis of AARD, such as SLE [3–5, 8]. Therefore, isolated anti-DFS70 antibodies represent a potentially important biomarker that can be used clinically to discriminate AARD from non-AARD patients in ANA-positive individuals.

At present, the introduction of new tests in clinical practice is hampered because of reimbursement challenges. In the daily routine, there is an excess of ANA requests. Some of them are due to the screening nature of the test, but there is also an increasing number of unnecessary repeat testing. [6]. From our experience, in most of the cases when an ANA result is positive but no specific antibody association is found, clinicians tend to order periodic ANA repetitions in patient follow-up. Moreover, in our jurisdiction this is not considered an isolated laboratory cost, since each request of ANA repetition is associated with an outpatient clinic visit just because of this positivity, generally with no symptomatic evidence and, most times, looking for an AARD that does not exist.

From our point of view, the identification of isolated anti-DFS70 antibodies can help classify patients and, because the presence on these antibodies is not related with AARD, would avoid unnecessary follow-up. In the present study, we determined if the implementation of a new algorithm containing anti-DFS70 antibodies is cost-effective through the reduction of unnecessary outpatient clinic visits generated by the suspicion of a potential AARD.

## Patients and methods

We evaluated samples from 181 patients, 157 females and 24 males, taken from our Autoimmune Serum Collection (Registration number at Instituto de Salud Carlos III, Spain: C.0001031) with a follow-up time of up to 10 years (mean of 4,75 years, SD: 5,41). These patients were suspected of having AARD and were positive for ANA, but with no evidence of a specific known ENA reactivity. The oldest serum sample from each patient was selected for analysis. Clinical records comprised reviews to confirm the primary disease, the cause of the first analytical request, and the evolution of all the diagnosis and treatment procedures, focusing especially on the number of outpatient clinic visits generated upon positive ANA result, and on the resolution of the initial AARD suspicion. All sera were ANA positive by IIF on HEp-2 cells. The main diagnoses were: SLE (*n* = 44), Sjögren`s syndrome (SS, *n* = 23), and non-AARD inflammatory disorders (*n* = 114) (Table 1).

**Table 1:** Diagnosis of the cohort and presence of anti-DFS70

Main diagnosis on the cohort	Number of patients (%)	Patients with anti-DFS70 antibodies
Systemic lupus erythematosus	44 (24)	1
Sjörgren syndrome	23 (13)	0
Systemic sclerosis	18 (10)	0
Dermatologic diseases	14 (8)	4
Arthritis	13 (7)	0
Rheumatologic diseases	10 (6)	0
Ophtalmologic diseases	8 (4)	5
Arthralgia	7 (4)	4
Arthrosis	6 (3)	3
Raynaud phenomenon	6 (3)	1
Hematologic diseases	5 (3)	2
Intestinal diseases	5 (3)	0
Neoplasms	5 (3)	0
Vasculitis	2 (1)	0
Others	15 (8)	3
Total	181	23

Rheumatologic diseases (fibromyalgia, dermatomyositis, antiphospholipid syndrome, rheumatic polymyalgia and ankylosing spondylitis)

Indirect immunofluorescence assay was performed using HEp-2 cells (BioSystems Diagnostics, Barcelona, Spain) used as secondary anti-human IgG conjugated to fluorescein isothiocynate (diluted 1/400; Dako, Gloostrup, Denmark). The screening dilution was 1/160 (followed by titrations of 1/320 1/640 1/1280 >1/1280). Reading and interpretation of the IIF patterns were done by an experienced immunologist on a Zeiss AxioPlan 2 IE microscope using a 40× objective.

The anti-dsDNA and anti-ENA assays (including the individual antigens RNP, Sm, Scl-70, Jo-1, Ro60, Ro52 and La) were performed by QUANTA Flash chemiluminescence immunoassays (CIAs, Inova Diagnostics, San Diego, USA), using the BIO-FLASH system (Biokit, Barcelona, Spain) following the procedure described previously [15, 16]. The QUANTA Flash assays used in this study were developed using native or recombinant antigens [15], coupled to the surface of paramagnetic beads. The reaction on BIO-FLASH is measured as relative light units (RLUs) by the BIO-FLASH optical system. The RLUs are proportional to the amount of isoluminol conjugate that is bound to the human IgG, which in turn is proportional to the amount of autoantibodies bound to the antigen on the beads.

All samples were also tested for the presence of anti-DFS70 antibodies by QUANTA Flash DFS70 CIA (Inova Diagnostics). This assay uses recombinant DFS70 (expressed in *E. coli*) coated onto paramagnetic beads and is designed for the BIO-FLASH instrument.

**Fig. 1:**
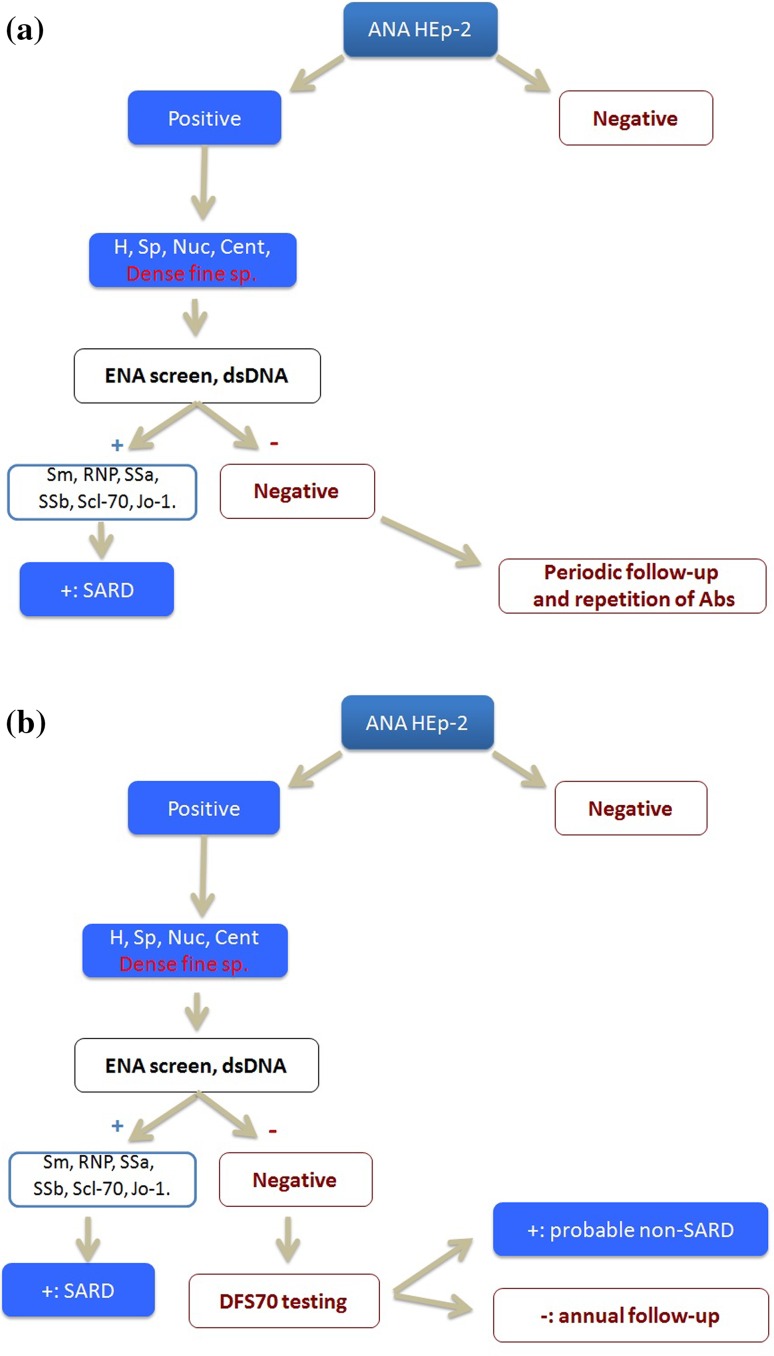
**a** Conventional algorithm used in HUMV (Spain). Abbreviations (*H*, *Sp*, *Nuc*, *Cent*, *Dense fine sp.*) refer to the different patterns found in ANA IIF test on HEp-2 cells: homogeneous, speckled, nucleolar, centromere and dense fine speckled. *SARD* Systemic autoimmune rheumatic diseases. **b** New algorithm including anti-DFS70 antibody detection. Abbreviations (*H*, *Sp*, *Nuc*, *Cent*, *Dense fine sp.*) made reference to the different patterns found in ANA IIF test on HEp-2 cells: homogeneous, speckled, nucleolar, centromere and dense fine speckled. *SARD* Systemic autoimmune rheumatic diseases

In this study, we compared the cost-effectiveness of two algorithms for the diagnosis of patients with AARD suspicion: The first algorithm is based on routine practice in our hospital (the Conventional Algorithm (Fig. 1a)), while the second algorithm includes the detection of anti-DFS70 antibodies as previously proposed by our group (the New Algorithm (Fig. 1b)) [17]. The conventional algorithm leads to unnecessary follow-up through the repetition of the entire antibody panel and the generation of specialist visits; in the new algorithm, an anti-DFS70 assay is added if ENA and dsDNA screening are negative upon a positive ANA result to classify these patients as potential non-AARD (if anti-DFS70 is positive) or as inconclusive result (if anti-DFS70 is negative). In the latter case, an annual follow-up is considered sufficient.

Data were statistically evaluated using SPSS software (version 22; IBM Corp.). Student’s *t* test was carried out to analyze difference between groups, and *p* values <0.05 were considered significant.

## Results

We observed that the presence of anti-DFS70 antibodies is not exclusive to the speckled pattern. The distribution of positive cases of anti-DFS70 antibodies in our cohort is spread between the speckled and homogeneous pattern to almost the same percentage in each pattern (Table 2).

**Table 2:** Anti-DFS70 antibody distribution depending on the IIF pattern

Anti-DFS70 presence	Homogeneus pattern	Speckled pattern	Centromere pattern	Total
DFS70−	93	54	11	158
DFS70+ (%)	16 (14.7 %)	7 (11.5 %)	0 (0 %)	23 (12.7 %)
Total	109	61	11	181

Secondly, none of the patients with an isolated positive anti-DFS70 antibody result developed AARD during the follow-up of the study. In these cases, ANA positivity could be explained by the presence of anti-DFS70 antibodies and no further actions would be necessary. Thus, these patients in our cohort would not have any advantage of either subsequent analytical determinations or outpatient clinic visits usually generated in addition. It is important to note that there was one patient with anti-DFS70 positivity who developed SLE, but it was the drug-induced form of the disease.

In the present study, we considered two criteria to compare costs: the laboratory ANA and follow-up testing, and the resulting clinic visits.

**Fig. 2:**
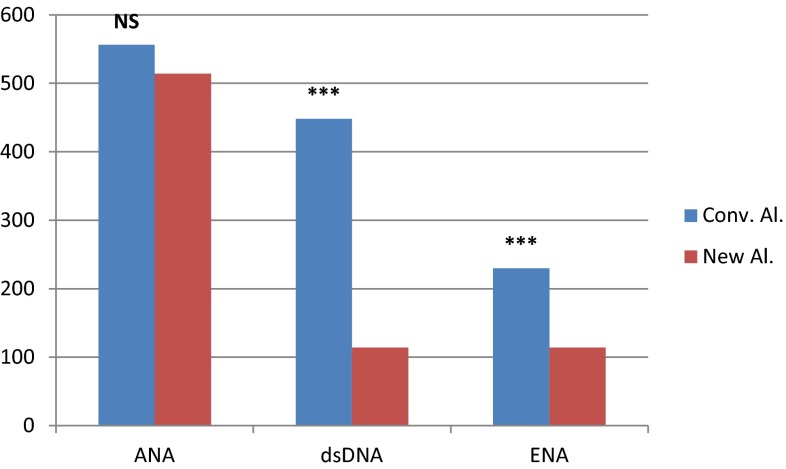
Differences in number of ANA IIF, anti-dsDNA and ENA determinations between the conventional and the new algorithm. (***p < 0.001; **p < 0.01; *p < 0.05; *NS* not significant)

When assessing laboratory costs in terms of ANA IIF testing, the conventional testing algorithm resulted in a total number of 556 tests compared to 514 tests using the new proposed algorithm (difference not statistically significant; *p* = 0.235). This means a small reduction in costs because we propose maintaining ANA IIF testing during the follow-up of the patients, even when there is no specific antibody associated with the ANA positivity (ENA, dsDNA or anti-DFS70). The reduction in number of tests was much more significant for anti-ENA (230 vs. 114 tests; *p* < 0.001) and anti-dsDNA antibodies (448 vs. 114 tests; *p* < 0.001) (Fig. 2). This is due to the periodic repetitions of these specific antibodies in the conventional algorithm, although in our patients they did not provide help in establishing a diagnosis.

**Fig. 3:**
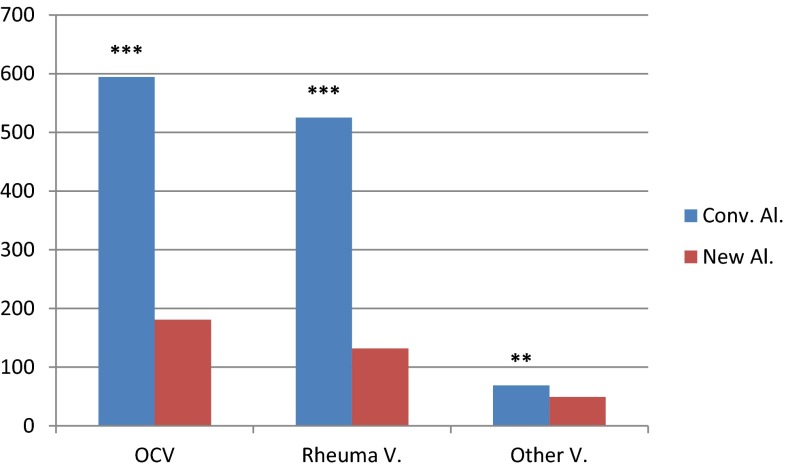
Differences in number of clinic visits between the conventional and the new algorithm. The total outpatient clinic visits (*OCV*) are split in rheumatologic visits (*Rheuma V*.) and the other visits (*Other V*.). (***p < 0.001; **p < 0.01; *p < 0.05; *NS* Not significant)

Using the new algorithm, the visits decreased by 70 % for the outpatient clinic (*p* < 0.001), by 75 % for rheumatologist (*p* < 0.001) and by 30 % for other specialties (*p* = 0.001) (Fig. 3). This reduction is due to the amount of unnecessary clinic visits generated during the follow-up of the patients using the conventional algorithm.

In addition to the pure economic issue, there are potential time and staff savings by reduction of outpatient clinic visits. Applying the new algorithm, there is no need for patients to be followed up as closely as they used to be by rheumatologists, who are the referral physicians for SARD patients in our hospital.

To translate all differences between the two algorithms to financial savings, we used the DRGs (diagnosis related groups) of our hospital during the years of follow-up of our cohort. We applied the cost of each ANA (IFI, anti-dsDNA and specific ENA) determination made, and the cost of the whole process that includes an outpatient clinic visit (of each specialist).

**Fig. 4:**
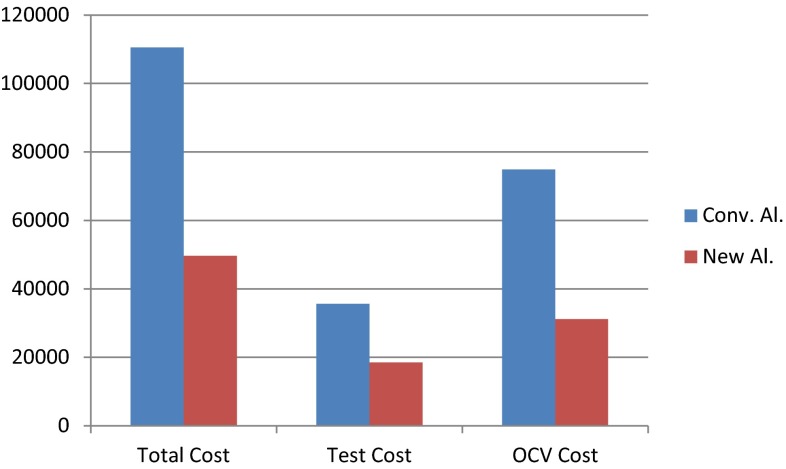
Comparison of total costs using the conventional and the new algorithm, showing costs of laboratory tests (*test cost*), costs of outpatient clinic visits (*OCV cost*), and total costs in Euros (*total cost*)

By applying the new proposed algorithm, we observed total savings of 17161.71 € in laboratory costs, and a saving of 43707.80 € in outpatient clinic visits (Fig. 4). In summary, the adoption of the new algorithm including anti-DFS70 antibody testing would result in a total cost saving of 60869.53 €.

## Discussion

We can estimate the basic savings in a cohort of only 181 patients more than 60,000 Euros. Thus, we are convinced that the adoption of the new algorithm including anti-DFS70 antibodies would be cost-effective. It may be a matter of discussion if it would be more convenient to go for a six-month follow-up instead of an annual follow-up; however, our data show that the savings would still be substantial.

Taking all of this into account, we can conclude that the use of anti-DFS70 antibodies in this preliminary study of patients with AARD suspicion was clearly cost-efficient. A prospective study needs to be initiated to obtain data of total savings per year and statistical power enough to make definitive changes in our hospital. This process must be established in collaboration with the principal services involved in the process, particularly the Rheumatology Department.
